# The Virtual City Paradigm^TM^ for Testing Visuo-Spatial Memory, Executive Functions and Cognitive Strategies in Children With ADHD: A Feasibility Study

**DOI:** 10.3389/fpsyt.2021.708434

**Published:** 2021-08-12

**Authors:** Benedetta Del Lucchese, Vittorio Belmonti, Paola Brovedani, Maria Celeste Caponi, Alexander Castilla, Gabriele Masi, Annalisa Tacchi, Mohamed Zaoui, Giovanni Cioni, Alain Berthoz

**Affiliations:** ^1^IRCCS Fondazione Stella Maris, Pisa, Italy; ^2^Laboratoire de Psychologie du Développement et de l'Éducation de l'Enfant (LaPsyDÉ, UMR CNRS 8240), Université de Paris, Paris, France; ^3^Laboratoire de Psychologie et de Neurosciences, Institut de Médecine Environnementale, Paris, France; ^4^Centre Interdisciplinaire de Recherche en Biologie, Collège de France, Paris, France; ^5^Department of Clinical and Experimental Medicine, University of Pisa, Pisa, Italy

**Keywords:** visuo-spatial memory, executive functions, navigation, ADHD, children, neurodevelopmental disorders

## Abstract

Navigation is a complex process, requiring target localization, route planning or retrieval, and physical displacement. Executive functions (EFs) such as working memory, inhibition and planning are fundamental for succeeding in this complex activity and are often impaired in Attention Deficit and Hyperactivity Disorder (ADHD). Our aim was to analyze the feasibility of a new ecological navigation task, the Virtual City paradigm™ (VC™) to test visuo-spatial memory and EFs in children with ADHD. Visuo-spatial short and working memory, inhibition and planning skills were tested with standardized tasks. The VC™, a new paradigm developed by our group, used the Virtual Carpet^TM^ technology, consisting of a virtual town with houses, streets and crossroads projected on the ground. It includes a motion capture system, tracking body movement in 3D in real time. In one condition, children were required to walk through the city and reach a sequence of houses. In the other, before walking, they had to plan the shortest path to reach the houses, inhibiting the prepotent response to start walking. The results show a good feasibility of the paradigm (feasibility checklist and ad hoc questionnaire), being ecological and motivating. VC™ measures of span positively correlated with visuo-spatial short and working memory measures, suggesting that VC™ heavily relies on efficient spatial memory. Individual subject analyses suggested that children with ADHD may approach this task differently from typically developing children. Larger samples of ADHD and healthy children may further explore the specific role of EFs and memory, potentially opening new avenues for intervention.

## Introduction

Spatial navigation is certainly one of the most complex neural functions in humans and one that is absolutely vital to everyday life. Retrieving locations and paths, planning routes to distant destinations, ascertaining one's location in space, drawing and reading maps, are all daily navigational tasks. A lack of navigation skills may impair one's ability to find things, reach targets, avoid obstacles, and return home. It may lead to complete dependence on others, or even to death, if experienced in a dangerous environment. In spite of a large amount of studies on navigation deficits in patients with neurological deficits (1–5), the availability of validated diagnostic tools for navigation disorders is still extremely limited. In addition, there are no studies assessing navigation in patients with neurodevelopmental disorders, as Attention Deficit and Hyperactivity Disorder (ADHD).

Traditionally, spatial navigation has been assessed by means of paper mazes, in manual space and not requiring locomotion. Only recently, novel tests for the assessment of navigation have been created and validated in adults and children (6, 7). The Magic Carpet is such a test and has been validated both in typically developing children and in children with cerebral palsy (8). It is derived from the Walking Corsi Test (9–11) and assesses locomotor navigation via the same procedure of the Corsi Block-Tapping Test for short-term visual-spatial memory, but translated from manual into locomotor space. By analyzing the errors made on the Magic Carpet (6, 8, 12) it has been possible to gain insight into the cognitive strategies used by different groups at different ages and to formulate hypotheses on the development of human navigation. However, the Magic Carpet did not allow measuring the kinematics of the trajectory, nor also the head direction as an index of gaze direction, as was done previously in the study of Belmonti (8) in typically developing children and children with Cerebral Palsy, capturing body motion during task execution.

The Virtual City paradigm (VC™) has therefore been developed in collaboration with the group in Paris of A. Berthoz [see (13)]. It is implemented using the Virtual Carpet™ experimental design (7, 14, 15), with the aim of assessing real locomotor navigation in a controlled laboratory space and under specific experimental conditions, allowing for grading of task difficulty and analysis of different neuropsychological functions. The nature of processes necessary for successfully completing such locomotor navigation tasks, such as egocentric and allocentric strategies, have been analyzed in the literature, both in adults (16–18) and in children (6, 8, 19, 20).

This new and ecological way of testing neuropsychological functions and cognitive strategies, in a motivating context, suitable for children with neurodevelopmental disorders, can be potentially highly informative for understanding executive functions (EFs) and memory in children with ADHD, for whom such functions are specifically challenging.

ADHD is a neurodevelopmental disorder with persistent inattention and/or hyperactivity/impulsivity, present in at least two life contexts, associated with significant social and academic impairment and with onset before 12 years of age (21). According to the Diagnostic and Statistical Manual of Mental Disorders – Fifth edition (DSM-5, 2013) (21), there are three ADHD presentations: predominantly inattentive, predominantly hyperactive/impulsive and combined. ADHD is one of the most prevalent childhood disorders with a worldwide prevalence of around 7%, with problems persisting into adulthood (22).

ADHD has a high heterogeneity at the clinical, genetic and neurocognitive levels (23). Children and adolescents with ADHD have been shown to consistently display differences in brain structure and function with respect to typically developing peers. Review of neuroimaging data indicate alterations prevalently in fronto-striatal, fronto-parieto-temporal, fronto-cerebellar and fronto-limbic networks, according to different neuropsychological and clinical phenotypes [for a review of neuroimaging studies see (24–26)]. At the cognitive level, ADHD is associated with a wide range of neuropsychological deficits, the most frequently reported being deficits in inhibition, memory, temporal discounting, decision making and timing, indicating that these constitute key cognitive domains, with EFs being heavily studied (27, 28). There are indications however that children and adolescents with ADHD may fall in distinct neuropsychological subgroups, displaying some but not all of the key cognitive deficits (29).

Among deficits in several cognitive areas, working memory, that is the function of actively holding in mind and manipulating information relevant to a goal, has received much attention (30, 31), also for tailoring rehabilitation (32). Visual-spatial short memory has been found to be more impaired than verbal short-term memory, and memory difficulties have been reported both at the level of storage and of active control/updating components in central executive tasks (33). Indeed, visual-spatial working memory may be thus a leading candidate endophenotype for ADHD.

Response inhibition is fundamental when alternative courses of thoughts or actions (planned or already initiated) have to be inhibited to allow the emergence of goal-directed behavior, and its deficit is associated with impulsive behaviors, a core DSM-5 diagnostic feature of ADHD. Reward-delay impulsivity has been explored with a meta-analytic method to examine differences in children and adolescents with and without ADHD (34), showing that youths with ADHD exhibited moderately increased impulsive decision-making compared to controls.

Deficits in planning abilities are also frequently reported in ADHD. A meta-analysis examined performance and latency measures in five tower planning task variants in 41 studies including ADHD, to calculate between-group effect sizes, and found moderate-magnitude planning deficits (35). Children with ADHD responded more quickly on planning tasks when compared to normal peers.

It has been also proposed that cognitive impairments in ADHD may result from both central controlled processes and more automatic information processes (36), with reciprocal functional interactions between subcortical regions and higher-order brain networks (37). The automatic processes, underpinned by dynamic subcortical circuits (including superior culliculus, pulvinar, and basal ganglia), may play a pivotal role in pathological distractibility of ADHD, representing “biological shortcuts,” which may bypass more complex systems, such as those involved in strategic planning (37, 38). Following this model, deficits in executive functions may be due, at least partly, to deficits in this automatic processing, leading to higher cognitive loads and limited resources available for EFs (39). Structural differences in subcortical structures in individuals with ADHD compared with those without this diagnosis may support this model.

Based on these considerations, the VC^TM^ paradigm was intended as a new and more ecological tool for assessing cognitive processes which are challenging for children with ADHD, as focused attention, memory, planning and inhibition, especially when they have to be recruited together as is the case in real-life situations.

The aim of this brief research report was to analyze, in a group of school-aged children diagnosed with ADHD, the feasibility of a navigation approach transferred to the VC^TM^ paradigm and its capacity to explore and measure the cognitive strategies used by these children during a visuo-spatial memory task. The feasibility study was thus specifically intended for this clinical population with significant impairments in these areas of cognitive functioning, which were also tested with classical neuropsychological tasks.

## Methods

### Subjects

The feasibility study included a clinical group of drug-naïve children with a diagnosis of ADHD, recruited in our third-level hospital of Child and Adolescent Neurology and Psychiatry. All participants underwent a multi-dimensional assessment, and diagnoses were made according to the DSM 5 (21), based on clinical history and a structured interview, Kiddie Schedule for Affective Disorders and Schizophrenia – Present and Lifetime version (K-SADS-PL) (40). The inclusion criteria were: (1) Diagnosis of ADHD; (2) Drug naïvité for stimulant treatment and any other pharmacotherapy; (3) Absence of intellectual disability; (4) Absence of comorbid conditions, except for Specific Learning Disabilities-SLD- (DSM 5); (5) Verbal intelligence of 85 or above (Wechsler Scales) (41, 42) to ensure full comprehension of the verbal instructions of the VC^TM^ paradigm; (6) Absence of any visual (non-corrected) or gait problems.

Twenty-two patients aged 7–13 years were recruited (mean 9;8 years; sd 1;9 years; males *n* = 17; 77%), all eligible to be included in the study. ADHD presentation was 77% combined (*n* = 17) and 23% inattentive (*n* = 5), 36% displaying comorbid SLD (*n* = 8). Mean verbal intelligence was 110.5 (sd 10.6). Demographic and clinical data for the entire sample of 22 participants is presented in Table 1.

**Table 1:** Demographic and clinical data of the ADHD sample.

**n**.	**Age (yrs;mo)**	**Sex**	**Adhd presentation**	**Specific learning disability**	**Intelligence (WISC-IV indices)**			
					**VCI**	**PRI**	**WMI**	**PSI**
1	7;11	M	Combined		104	98	82	68
2	7;3	F	Combined		116	93	82	56
3	9;6	M	Combined		120	106	121	123
4	8;0	M	Combined		100	91	61	53
5	8;2	M	Combined		104^*^	96^**^	NA	NA
6	9;5	M	Combined		120	93	97	94
7	7;10	M	Combined		90	80	79	82
8	8;11	M	Combined	Yes	108	100	94	94
9	9;8	F	Combined	Yes	98	89	79	94
10	13;8	M	Combined	Yes	122	108	112	74
11	12;10	M	Combined	Yes	96	102	82	94
12	8;5	M	Combined		100	91	70	85
13	8;0	F	Combined		112	126	94	79
14	10;7	M	Combined		114	124	103	118
15	9;3	M	Combined		132	113	94	88
16	8;9	F	Combined	Yes	114	100	91	71
17	10;7	M	Combined		106	124	94	79
18	12;8	M	Inattentive	Yes	112	119	103	123
19	13;1	M	Inattentive		108	104	94	88
20	12;3	M	Inattentive		120	122	103	79
21	8;8	F	Inattentive	Yes	128	91	85	82
22	10;3	M	Inattentive	Yes	108	91	82	109

*VCI Verbal Comprehension Index; PRI Perceptual Reasoning Index; WMI Working Memory Index; PSI Processing Speed Index;*

* *Verbal Intelligence quotient and*

** *Performance Intelligence quotient at WPPSI-III at 6;8 years; NA not applicable*.

This study complied with the Declaration of Helsinki and was approved by the Regional Pediatric Ethical Committee (n.175/2019). Parents and children signed a written consent form (for children, in a child friendly format).

### Procedures and Measures

The experimental design was divided into two assessments administered to each child: the VC^TM^ paradigm and neuropsychological tasks, both testing visuo-spatial memory and EFs. The VC^TM^ paradigm and neuropsychological tests were carried out at different times of the same day or on two different days (no longer than a week apart), in order to reduce the fatigue effect as much as possible. Order of assessments was randomized with half of the participants starting with the VC^TM^ paradigm and the other with the neuropsychological evaluation, in the majority of cases. Duration of the entire VC^TM^ paradigm ranged from 40 to 50 min in a single session although for some children, due to variability in collaboration, duration could be longer. Subsequently, the psychologists (BDL and MCC) who administered the task, filled out a feasibility VC^TM^ questionnaire created ad hoc. The duration of the neuropsychological assessment was 1 h on average in one single session but varied again as a function of degree of collaboration.

The experimental set up and the procedures were the following:

#### The Virtual City Paradigm™

**Figure 1:**
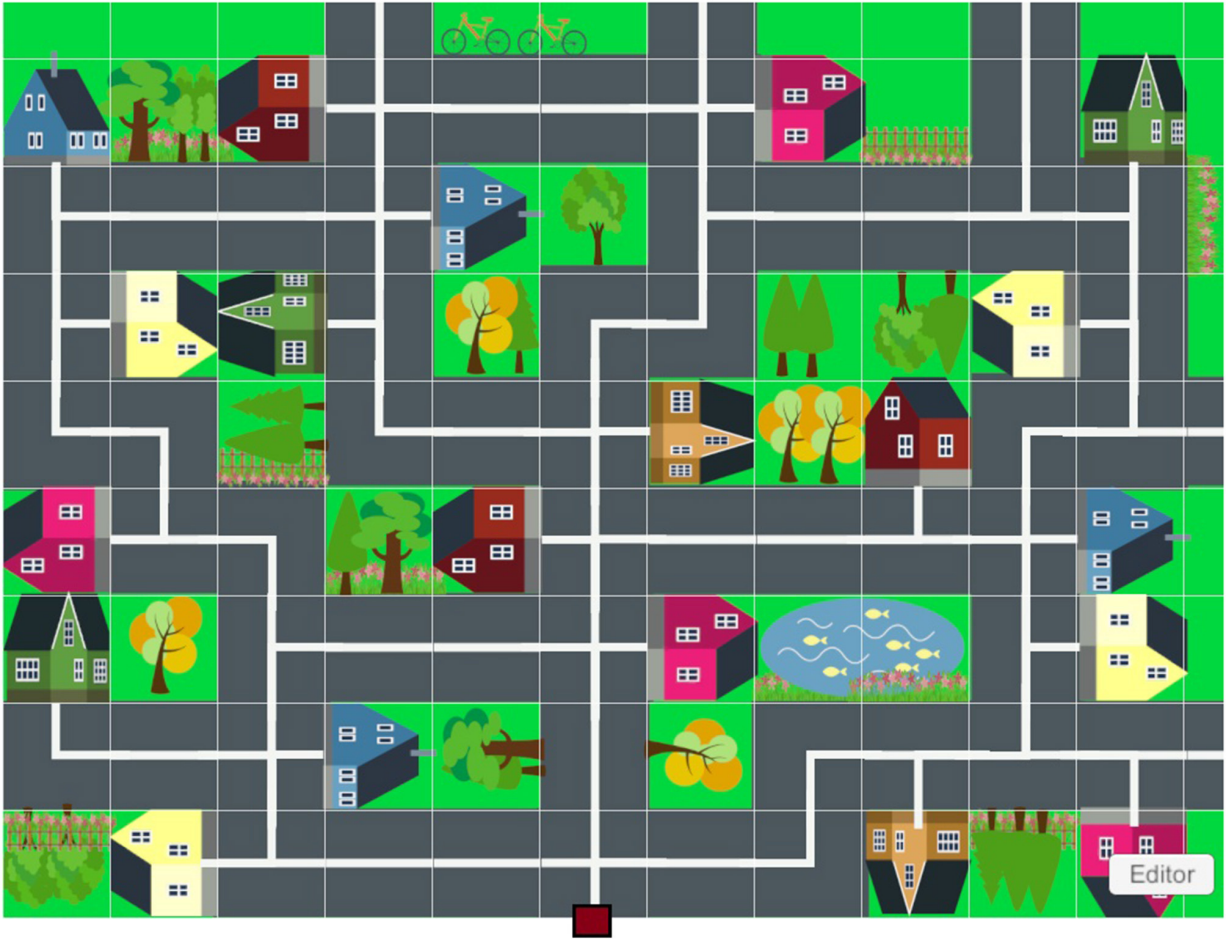
VC^TM^ projection on the ground. The red square indicates the starting position.

The VC™ is a projected virtual town on the floor, consisting of 20 houses, street lanes and crossings (Figure 1), created on Unity 5.5.1^©^ platform. Two projectors were installed and connected to a computer so as to project the town on an off-white carpet (2.6 m × 3.2 m) in a dark laboratory space. The child had to move around the virtual town to reach the houses which flickered (the targets). Houses flickered either in a sequence, or all together. For tracking the trajectory of the child, the motion capture system (HTC^®^ Vive and Steam^©^ software), included two handheld three-dimensional space (3D) motion sensors applied one on the head (fixed on a bike helmet worn by the child) and one on the trunk (fixed on a belt worn by the child) (see (13)) and two infrared cameras allowing tracking of body movement in 3D in real time (see videos in the Supplementary Material).

The VC^TM^ paradigm included three different conditions in which the number of houses to be reached (span level), the sequence order, flicker duration, and the instructions varied.

1 City Pointing: While keeping the starting position, the child was asked to point (with a laser pointer) each house as it flickered (for 2.5 s). The sequence of flickering houses was randomized and the houses' order was set so that no contiguous houses flickered in a sequence. This procedure allowed assessing efficacy of visual search abilities in a large space and visuo-spatial span. If the child correctly pointed to at least 80% of the houses, the other conditions were administered.
2 City Following: A given number of houses was made to flicker in sequence. The child was asked to remain in the starting position and observe each house as it flickered (for 2.5 s). Then the child was asked to walk on the streets to reach each house in the same order he/she had seen them flickering. The sequences were randomized and the houses' order set with a mathematical algorithm to ensure both easy sequences (the houses are near to each other and not too many rotations are needed to reach the next one) and some difficult ones (i.e., more distant houses and more rotations). There was a maximum of five span levels (from the starting level of two houses for all subjects up to a level of six houses). Criterion for success on any given level was three out of five trials correct and in case of failure, five additional trials for the same level were presented before proceeding with the third condition. Similarly to the Corsi Block Tapping test, a span measure was obtained, but for this paradigm it was the longest sequence reached by the subject (even if the three out of five criterion was not met).
3 City Planning: The child was asked to observe the houses that were flickering simultaneously while keeping the starting position, and then to walk on the streets to reach the houses he/she had seen flickering. The specific instruction was to plan the shortest path. There was a maximum of three span levels (from a span of two to a span of four) each with 10 trials, with the starting span level being the span level reached in the second condition. Flickering duration for each span level was respectively 7.4, 11.3, and 13.1 s.

The cognitive strategies needed to complete the VC^TM^ tasks could be the following: a first encoding phase in which the subject mentally encoded the spatial distribution of the houses and eventually the temporal sequence of their presentation. This encoding may be perturbed in ADHD due to a deficit in selective attention and/or spatial memory. For this reason, a control condition was added (City Pointing), to ensure that children do indeed pay attention to all houses in the town as they flicker; a second recall phase in which before starting the task, the subject had to mentally rehearse the encoded representation of the flickering houses' spatial distribution and to generate the trajectory. Both phases imply spatial short- and long-term memory and inhibition, intended, the latter, as the capacity to inhibit the prepotent response to start walking in the town before having generated a trajectory or the shortest path as in the City Planning condition; finally, when the subject navigated the town, he/she needed to update the mental trajectory of the houses he/she had generated. That is, he/she had to represent the position of the houses relative to his actual position in the town and no longer the one relative to the starting position in which he/she had originally encoded them. This phase could tax the updating component of spatial memory (working memory).

In addition to the span measure, the VC^TM^ paradigm provides kinematics data on the movement trajectory of each subject. In particular, the HTC^©^ Vive system and Steam^©^ software allows both to generate the target positions (i.e., the houses) in the virtual environment (calibration procedure) and to record the trajectories of each child during navigation. The calibration procedure was performed by the psychologist (BDL) who positioned herself over each target house following a standard order, enabling to configure the global navigational array and to set the houses' positions in a cartesian coordinate system by triggering the 3D motion sensor.

To record the trajectory of the children, the system detected the locomotion during the experimental sessions and computed, for specific time frames (in ms), head and trunk sensor positions on X, Y, and Z axes, and rotation angles with respect to the X, Y, Z axes direction. These data were treated using Matlab 2021 to yield parameters such as trunk and head position and rotation in the horizontal plane, trunk and head velocity, acceleration, and stops during the trajectory. Further details on automatic kinematic data analysis are reported in (13).

#### Neuropsychological Assessment

Visuo-spatial short-term memory/working memory tasks in the reaching space included the Corsi Block Tapping task forward and backward (43) and a computerized block tapping task, the Spatial Span Task (CANTAB^®^) (44). The span measure was the longest sequence correctly retrieved. The Digit span WISC-IV subtests-forward and backward- served as a control verbal measure of spatial memory. Parents and children filled out a pilot questionnaire on everyday visuo-spatial and navigation abilities (Santa Barbara Sense of Direction Scale-Parent and Child Version: p-CBSOD and c-CBSOD) adapted by Murias et al. (45) (see Supplementary Material 1).

The Stop Signal Task (CANTAB^®^) (44) was administered as a measure of response inhibition. It is a go-no-go task adapting the time interval between the go stimulus and the stop stimulus to the performance of the subject providing as the outcome measure, the estimate of time during which an individual can successfully inhibit the response 50% of the time. The Tower of London (46) was administered as a measure of planning expressed in terms of total decision time, execution time and number of rule violations. As an ecological measure of EFs, parents filled out the Behavior Rating Inventory of Executive Function - Second Edition (BRIEF-2) (47) on their children's abilities for inhibition, working memory, monitoring and self-monitoring, shift, planning and emotional regulation.

#### Feasibility Assessment

The feasibility of the VC^TM^ paradigm was investigated with two measures, an *ad-hoc* questionnaire on acceptability and usability filled out by the two experimenters (BDL and MCC) and a feasibility checklist. The questionnaire (see Supplementary Material 2), conforming to the standard definitions of usability (48–50) and acceptability (51, 52) [for a review study see (53)], consisted of 14 questions ranked on a 5-point Likert scale (1 most negative, 5 most positive). The feasibility checklist with criteria for success, based on a literature review (see Supplementary Material 2, Table 1), consisted of nine outcome measures grouped in four areas specific for the VC^TM^ (accessibility, training motivation, technical smoothness, and training compliance) and 5 for the entire study design and procedures (participation willingness, participation rates, loss to follow-up, assessment timescale and assessment procedures).

## Results

### Feasibility Analyses

Feasibility questionnaire data and checklist measures were available for 21/22 subjects. Feasibility questionnaire results for usability and acceptability revealed a prevalence of positive responses, indicating a satisfactory feasibility of the VC™ paradigm. For usability (6 questions), there were 74/126 responses graded as 5 and 29/126 as 4. For acceptability (8 questions), there 73/168 graded as 5 and 44/168 as 4.

Feasibility criteria were met for all measures both for the VC™ (accessibility 91%; compliance 91%; technical smoothness 32%; motivation 14%) and for the entire study design and procedures (participation willingness 95%; participation rates 4%; missing data: VC™ and neuropsychological assessment 13%; time scale 91%; procedure 91%).

### VC™ Span Level and Neuropsychological Measures

The VC™ span level and neuropsychological measures were available for 18 out of 22 subjects due to 1 drop-out because parents refused to continue the study, 1 to technical sensors problems, and 2 for failure to complete the entire VC™ in a single session. Missing data (either Tower of London or WISC-IV digit span) concerned three subjects.

Group data will be presented first and then data from two 10 year-old children with ADHD deemed exemplary. A typically developing 10 year-old child served as a comparison subject.

Statistical analyses were computed with RStudio version 2020 for Windows (www. R-project.org). Preliminary Spearman correlation analyses were computed between the VC™ span and neuropsychological measures. The span level of the City Following condition, intended as the longest sequence reached (but not passed), was compared with the raw data of the different neuropsychological measures (Corsi Span, CANTAB^©^ Spatial Span, CANTAB^©^ Stop Signal, Tower of London, BRIEF-2) and with the standard WISC-IV Digit Span scores.

As expected, there was significant correlation between the VC™ span level and both the Corsi forward (*r* = 0.67, *p* = 0.002) and backward spans (*r* = 0.60, *p* = 0.008). In addition, there was a significant positive correlation between the VC™ span and the backward digit span (*r* = 0.57, *p* = 0.01). Age correlated significantly with the VC™ span level (*r* = 0.70, *p* = < 0.001). A significant negative correlation was found between c-SBSOD and VC^TM^ span level (*r* = −0.70, *p* = 0.001). No other significant correlation was observed with other neuropsychological test measures (Tower of London and CANTAB^©^ span and inhibition) and questionnaire measures (BRIEF-2, p-SBSOD).

### Individual VC™ Trajectories and Neuropsychological Data

**Figure 2:**
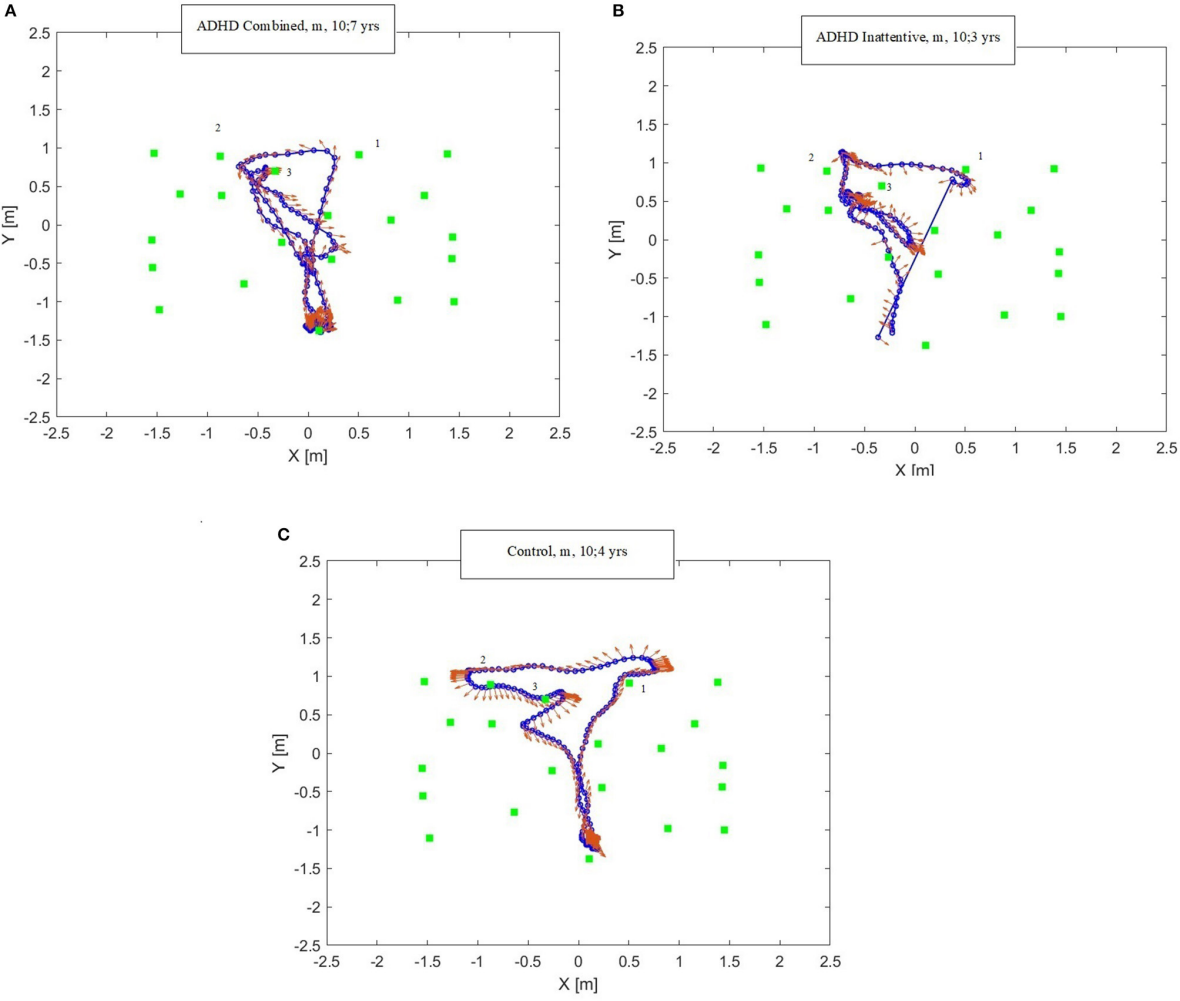
**(A–C)** Trajectories at the VC^TM^ paradigm of two children with ADHD and one control child. Blue lines and circles: motion trajectories; Red arrows: head direction with respect to the trajectory; Green squares: house position with sequence number on top. X and Y axes indicate the sensors' position in the navigational array (meters). **(A)** trajectory of a combined type ADHD child, male, aged 10;7 years; **(B)** trajectory of an inattentive type ADHD child, male, aged 10;3 years; **(C)** trajectory of a control subject, male, aged 10;4 years.

Based on trajectories analyses, a qualitative description of the behavior during VC™ performance is presented for two children with ADHD (subject 22, Inattentive and subject 17 Combined, Table 1), and the comparison subject. Figure 2 compares the trajectories of the same sequence (span level 3, trial 3) in the City Following condition, where the child is asked to reach three houses flickering in an easy sequence.

In Figure 2A, the child with Combined ADHD performed the trial correctly by reaching the 3 target houses in the right order. However, he reached the first and second target houses, then stopped, not remembering the exact position of the third target house. He therefore returned to the starting position, looked around (as indicated by the red arrows), then he presumably remembered the position of the third target house and headed toward it. In Figure 2B, the child with Inattentive ADHD failed the task. The child started from the initial position and correctly reached the first and second houses. He then reached a wrong house, then stopped, looked around, understood that he had failed and thus proceeded to reaching another (incorrect) house. From Figure 2B, this child's head movements, shown by red arrows, indicate a high distractibility of the subject, given his frequent deviation from the trajectory and they do not predict the following movement directions. Figure 2C shows that the comparison child reached the target houses in the right order with a linear locomotion trajectory. The head movements did not deviate from the path when linear, while they were anticipatory when body rotations were necessary, predicting the following movement directions. Neuropsychological assessment data of the two children with ADHD and the comparison subject revealed some important qualitative differences. They concern not only visuo-spatial memory abilities (Corsi span forward and backward), but also EFs, a core deficit of ADHD children. Specifically, with regards to the parent report questionnaire BRIEF-2, the cognitive regulation abilities (Cognitive Regulation Index) were much poorer in the children with ADHD than in the control, with T scores in the clinical/borderline range. Tower of London performance indicated significant difficulties only in the Inattentive presentation. Such skills could be crucial for carrying out the task, and include planning, working memory and self-monitoring. CANTAB^©^ and SBSOD (child and parent report) data were not available for the comparison subject and thus are not presented.

## Discussion

### Feasibility

The primary aim of the study was to evaluate the feasibility of the VC paradigm^TM^ for assessing visual-spatial memory and EFs in a navigation task in children with ADHD. The results from the *ad-hoc* feasibility questionnaire indicated satisfactory usability and acceptability. Regarding usability, the device could be used efficiently with no need for external technical support, with intuitive hardware and software instructions, the sensors being non-invasive and the entire device not posing any danger to the child. Concerning acceptability, the VC^TM^ proved to be a motivating and playful task for children, potentially informing clinical practice, recruiting different cognitive strategies than the neuropsychological tests presented in peri-personal space. The VC™ appeared to be a more ecological assessment measure as it investigates the skills required in daily life. However, some technical issues with the motion sensors limited correct data acquisition. This could be due to the high level of hyperactivity combined with the sensors' high sensitivity, both interfering with the position acquisition by the cameras. The feasibility checklist highlighted a good compliance, as the great majority of the subjects performed the entire task and within the designated time frame. The children were also very motivated and reported a limited effort in carrying out the task. Concerning the feasibility of the entire study design and procedures, the participation rate was extremely high, as none of the participants except one dropped out of the study.

### Correlations Between Virtual City™ Span and Neuropsychological Measures

Significant associations were found between VC^TM^ span -Following condition- and verbal and visuo-spatial memory abilities. A larger correlation was found between the VC ^TM^ span and the Corsi Block Tapping test. No associations were found between the VC^TM^ span (Following condition) and EF measures differently than expected from the literature (6, 8). The VC™ span was the sole measure to be analyzed, while other available parameters such as head deviation from the trunk, latency and kinematic parameters may offer new insights into the role of EFs. Furthermore, the EF measures chosen may not have been sensitive enough. The negative correlation between the Child SBSOD questionnaire and the VC^TM^ span was unexpected. Better perception for one's spatial orientation abilities was associated with lower VC^TM^ span. This could be due to difficulty in fully understanding the questions, as well as to a reduced awareness of one's own deficits.

### Performance Differences in ADHD Subjects Compared With the Control Child

The trajectory analyses of ADHD and control subjects reveal some qualitative differences in spatial navigation behavior which may be associated with the deficits displayed by children with ADHD.

Although the child with combined ADHD performed the sequence correctly, the locomotor pathway was non-linear. In fact, this child went back to the starting point possibly to rehearse the trajectory previously encoded. This suggests that he recruited an egocentric storing strategy less functional than an allocentric one. This return-to-start behavior has been described in adults (54) in a “virtual starmaze” task and accounted for as “a mixed strategy.” During navigation, sensory stimuli can be encoded in spatial reference frames centered on the sensory organs (egocentric) or in an allocentric reference frame, with allocentric spatial encoding strategy introducing a substantial computational simplification, acquired later in childhood and probably subsumed by EFs (7). Since executive dysfunction is one of the core deficits of ADHD, these children may have difficulties in activating an allocentric strategy to store the targets. The child with inattentive ADHD showed the worst performance, being highly distractible, failing the sequence, following a linear path (he did not return to the starting point), with head and trunk not moving in the same directions.

Given the novelty of this complex navigation paradigm, tapping processes beyond executive functions, it is premature to interpret the preliminary results in terms of specific models or hypotheses on attentional/executive dysfunctions in ADHD. Further analyses on the planning trajectories and on the pattern of responses of typically developing children could provide insights on the role of automatic processes which could be preponderant in approaching this task in ADHD but also in younger children.

Infact, no age-matched control group was recruited for this study. However, as already highlighted, this is a feasibility study aimed at analyzing usability and acceptability of a new way of testing cognition in navigation in a clinical population with significant impairments in cognitive functions tapped in the VC^TM^ paradigm. A study on typically developing children will be conducted, matched to a larger group of children with ADHD for analyzing if there are specific patterns of behavior which characterize this clinical population, as suggested by the preliminary trajectories' analyses. To better understand the cognitive processes involved in the VC^TM^ task, further investigations will be necessary, taking into account parameters other than span such as decision time, head deviation from trajectory, to name the most relevant that have been studied in other navigational tasks. These indicators could clarify the role and nature of EFs that did not clearly emerge in this feasibility study, but are certainly involved in such a challenging navigational task. Further neuropsychological assessments could be advantageous as to allow disentangling specific cognitive processes which may be pivotal for understanding how children approach this ecological yet complex task.

## Data Availability Statement

The original contributions presented in the study are included in the article/Supplementary Material, further inquiries can be directed to the corresponding author/s.

## Ethics Statement

The studies involving human participants were reviewed and approved by Regional Pediatric Ethical Committee (n.175/2019), Tuscany region, Italy. Written informed consent to participate in this study was provided by the participants' legal guardian/next of kin. Written informed consent was obtained from the individual(s), and minor(s)' legal guardian/next of kin, for the publication of any potentially identifiable images or data included in this article.

## Author Contributions

BDL: methodology, investigation, resources, data curation, writing—original draft, and writing—review and editing. VB: conceptualization, methodology, formal analysis, and writing—review and editing. PB: methodology, formal analysis, supervision, writing original draft, and writing—review and editing. MCC: formal analysis, investigation, resources, and writing—review and editing. AC: methodology, software, validation, data curation, and writing—review and editing. GM: methodology, supervision, resources, and writing—review and editing. AT: methodology, resources, and writing—review and editing. MZ: resources, data curation, visualization, and formal analyses. GC: conceptualization, supervision, writing, review and editing, and funding acquisition project administration. AB: methodology, conceptualization, formal analysis, supervision, writing, and review and editing. All authors contributed to the article and approved the submitted version.

## Conflict of Interest

The authors declare that the research was conducted in the absence of any commercial or financial relationships that could be construed as a potential conflict of interest.

## Publisher's Note

All claims expressed in this article are solely those of the authors and do not necessarily represent those of their affiliated organizations, or those of the publisher, the editors and the reviewers. Any product that may be evaluated in this article, or claim that may be made by its manufacturer, is not guaranteed or endorsed by the publisher.
