# Supplementary material for: The Virtual City ParadigmTM for Testing Visuo-Spatial Memory, Executive Functions and Cognitive Strategies in Children With ADHD: A Feasibility Study
Source: Front Psychiatry. 2021 Aug 12;12:708434. doi: 10.3389/fpsyt.2021.708434 (PMC8406804; doi:10.3389/fpsyt.2021.708434)
Supplement: Supplementary file 1 [file Data_Sheet_1.docx]

**Supplementary Material 1.** Child and Parent version of the Santa Barbara Sense of Directions SBSOD (Murias et al., 2017)

CHILD MODIFIED SBSOD

1. I am very good at giving directions to help people find places or things

Strongly agree 1 2 3 4 5 6 7 Strongly disagree Unsure

3. I am good at guessing how far away things are

Strongly agree 1 2 3 4 5 6 7 Strongly disagree Unsure

4. I can imagine directions

Strongly agree 1 2 3 4 5 6 7 Strongly disagree Unsure

5. I like to use north, west, east, south when I find places

Strongly agree 1 2 3 4 5 6 7 Strongly disagree Unsure

6. I get lost easily

Strongly agree 1 2 3 4 5 6 7 Strongly disagree Unsure

7. I like to read maps

Strongly agree 1 2 3 4 5 6 7 Strongly disagree Unsure

8. It is hard for me to follow directions to new places

Strongly agree 1 2 3 4 5 6 7 Strongly disagree Unsure

9. I am good at reading maps

Strongly agree 1 2 3 4 5 6 7 Strongly disagree Unsure

10. I don’t usually remember how to get to places

Strongly agree 1 2 3 4 5 6 7 Strongly disagree Unsure

11. I don’t like giving people directions

Strongly agree 1 2 3 4 5 6 7 Strongly disagree Unsure

12. I like it when to know where I am and where I’m going

Strongly agree 1 2 3 4 5 6 7 Strongly disagree Unsure

13. I can remember how to get somewhere after going there once

Strongly agree 1 2 3 4 5 6 7 Strongly disagree Unsure

14. I can imagine where things are in my environment

Strongly agree 1 2 3 4 5 6 7 Strongly disagree Unsure

15. I am good at knowing when I’ve been to a place before

Strongly agree 1 2 3 4 5 6 7 Strongly disagree Unsure

16. I can tell the difference between left and right

Strongly agree 1 2 3 4 5 6 7 Strongly disagree Unsure

18. When I am lost, I cannot find my way back

Strongly agree 1 2 3 4 5 6 7 Strongly disagree Unsure

19. I like visiting new places

Strongly agree 1 2 3 4 5 6 7 Strongly disagree Unsure

20. I visit new places often

Strongly agree 1 2 3 4 5 6 7 Strongly disagree Unsure

SBSOD – PARENT VERSION

After reading each statement carefully, circle the number that indicates how well each statement

applies to your child. If you are unsure or have not observed these behaviors in your child,

circle unsure.

1. My child is very good at giving spatial directions

Strongly agree 1 2 3 4 5 6 7 Strongly disagree Unsure

2. My child has poor memory for where he/she left things

Strongly agree 1 2 3 4 5 6 7 Strongly disagree Unsure

3. My child is very good at judging distances

Strongly agree 1 2 3 4 5 6 7 Strongly disagree Unsure

4. My child’s “sense of direction” is very good

Strongly agree 1 2 3 4 5 6 7 Strongly disagree Unsure

5. My child tend to think of environment in terms of cardinal directions (N, E, S, W)

Strongly agree 1 2 3 4 5 6 7 Strongly disagree Unsure

6. My child gets lost very easily in a new city

Strongly agree 1 2 3 4 5 6 7 Strongly disagree Unsure

7. My child enjoys reading maps

Strongly agree 1 2 3 4 5 6 7 Strongly disagree Unsure

8. My child has trouble understanding spatial directions

Strongly agree 1 2 3 4 5 6 7 Strongly disagree Unsure

9. My child is very good at reading maps

Strongly agree 1 2 3 4 5 6 7 Strongly disagree Unsure

10. My child doesn’t remember routes very well while traveling in a car

Strongly agree 1 2 3 4 5 6 7 Strongly disagree Unsure

11. My child doesn’t enjoy giving directions

Strongly agree 1 2 3 4 5 6 7 Strongly disagree Unsure

12. It is not important to my child to know where he or she is

Strongly agree 1 2 3 4 5 6 7 Strongly disagree Unsure

13. My child can usually remember new route after he or she has traveled it only once

Strongly agree 1 2 3 4 5 6 7 Strongly disagree Unsure

14. My child doesn’t have a very good “mental map” of his or her environment

Strongly agree 1 2 3 4 5 6 7 Strongly disagree Unsure

15. My child is good at recognizing familiar places

Strongly agree 1 2 3 4 5 6 7 Strongly disagree Unsure

16. My child is good at discriminating between left and right

Strongly agree 1 2 3 4 5 6 7 Strongly disagree Unsure

17. My child gets lost easily in familiar environments that he or she visits at least once a month

Strongly agree 1 2 3 4 5 6 7 Strongly disagree Unsure

18. When my child gets lost, it is difficult for him or her to find the way back

Strongly agree 1 2 3 4 5 6 7 Strongly disagree Unsure

19. My child likes to explore new environments

Strongly agree 1 2 3 4 5 6 7 Strongly disagree Unsure

20. My child frequently visits new or unfamiliar environments (e.g., a park that he or she has

never been to before)

Strongly agree 1 2 3 4 5 6 7 Strongly disagree Unsure
