# Supplementary material for: The Virtual City ParadigmTM for Testing Visuo-Spatial Memory, Executive Functions and Cognitive Strategies in Children With ADHD: A Feasibility Study
Source: Front Psychiatry. 2021 Aug 12;12:708434. doi: 10.3389/fpsyt.2021.708434 (PMC8406804; doi:10.3389/fpsyt.2021.708434)
Supplement: Supplementary file 2 [file Data_Sheet_2.docx]

**Supplementary Material 2**

**VIRTUAL CITY PARADIGM™: FEASIBILITY MEASURES**

**Supplementary Material 2.
Questionnaire:** Virtual City Paradigm^TM^ feasibility questionnaire.

Please complete with examiner, participant and testing information

1. Name and title of examiner ______________________________________________________________________________
2. Participant’s ID code and age

_________________________________________________________________________________

1. Date of testing

_________________________________________________________________________________

1. Indicate order of testing (1 or 2)


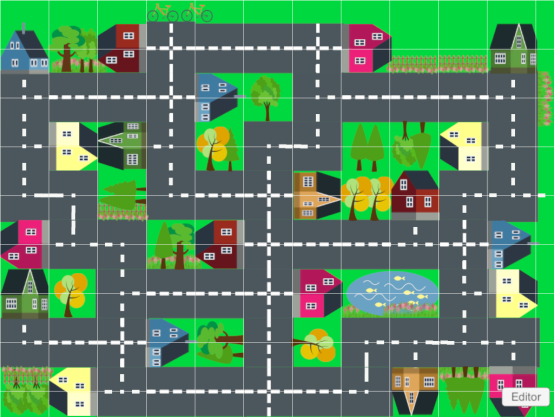

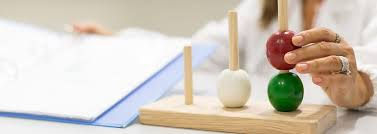
Virtual City Paradigm™ Neuropsychological testing


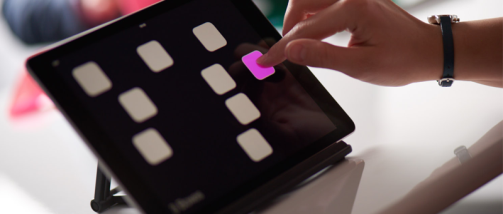


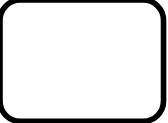


1. Length of time needed to complete the Virtual City Paradigm™:

- 10-30 minutes
- 30-45 minutes
- 45 minutes- 1 hour
- Greater than 1 hour

1. Total time needed for testing (Virtual City + Neuropsychological testing)

- 30-45 minutes
- 45 minutes- 1 hour
- 1 hour – 90 minutes
- 90 minutes – 2 hours
- Greater than 2 hours

Please answer the following questions on a scale from 1 to 5: 1 not at all - 5 very much (satisfied); 1 never - 5 all the time (how often)

*Please be aware that for some items the scale is reversed*

Usability

1. In general, relative to this specific participant, how satisfied are you with the Virtual City Paradigm™ (in terms of software, hardware and sensors- taken as a whole-)?

Not at all Very much

1

2

3

4

5

1. Your current knowledge of the system’s functioning (software, hardware and sensors -taken as a whole-) is sufficient for use with this specific participant?


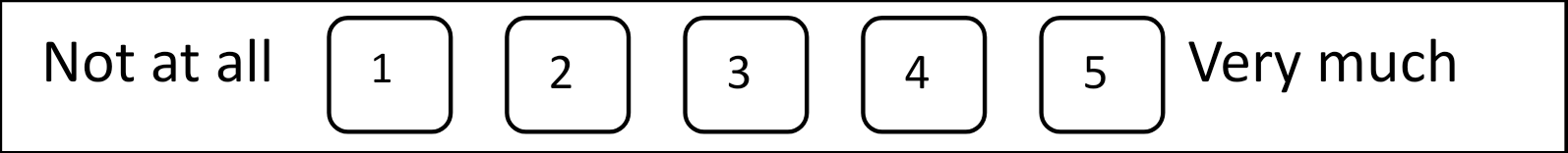


1. Do you believe that the system is suitable for this specific participant (in terms of carpet’s size, sensors’ size and wearability)?


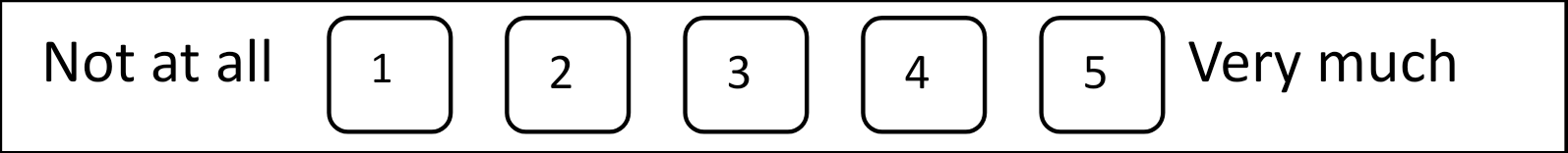


If not at all , please suggest modifications to the system: ___________________________________________________________________________________________________________________________________________________________________________________________________________________________________________________________

1. Were you able to achieve the goals you set for this specific participant?

Not at all Very much

1

2

3

4

5

1. How dangerous is it to use this system with this specific participant?


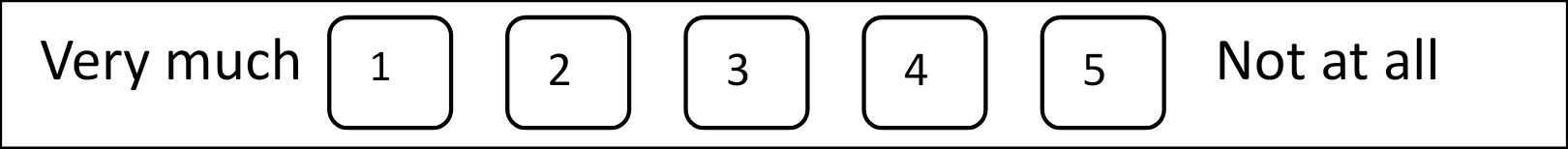


1. How easy was it to set the hardware for this specific participant?

Not at all Very much

1

2

3

4

5

Acceptability

1. How easy was it to use this system with this specific participant?

Not at all Very much

1

2

3

4

5

1. Did the Virtual City Paradigm™ allow you analyze the skills that you intended to measure for this specific participant?

Not at all Very much

1

2

3

4

5

1. How often did you have to interrupt the paradigm and provide a greater number of rests than established with this specific participant due to reduction in motivation or eccessive mental or physical fatigue on the part of the participant?

Never All the time

1

2

3

4

5

1. How often did you need to interrupt the paradigm with this specific participant due to technical issues arising during the system’s utilization?

All the time Never

1

2

3

4

5

1. Do you believe the system needs to be modified to address this specific participant’s needs?

Very much Not at all

1

2

3

4

5

1. If so, what needs to be modified and why?

___________________________________________________________________________________________________________________________________________________________________________________________________________________________________________________

1. Do you believe that the system’s data report is useful in clinical terms?

Not at all Very much

1

2

3

4

5

1. Please illustrate why or why not

___________________________________________________________________________________________________________________________________________________________________________________________________________________________________________________

1. Would you use this system as an intervention paradigm for this specific participant?

Not at all Very much

1

2

3

4

5

1. Has the Virtual City paradigm™ data stimulated your thinking on aspects of behavior that had not emerged from clinical evaluation?

Not at all Very much

1

2

3

4

5

**Supplementary Material 2.**

**Table 1. Checklist.** Feasibility criteria and measures based on literature parameters. The feasibility checklist consists of 9 outcome measures, 4 relative to the Virtual City paradigm™ and 5 for the entire study design and procedures.

|  | **Feasibility criteria** | **Definition** | **Feasibility question** | **Measurement** | **Feasibility criterion for success** |
| --- | --- | --- | --- | --- | --- |
| **Feasibility of Virtual City ™ assessment** | **Accessibility** | Intelligibility of instructions for performing the paradigm | Do participants understand aims and rules of the paradigm as provided by the standardized verbal instructions of the experimenter? | Record of number of participants not needing more than 2 instructions or clarifications | At least 80% of participants not needing more than 2 clarifications |
|  | **Virtual City™ compliance** | Duration of the assessment | Do participants perform the entire task within an hour time frame and manage to complete the entire Virtual City paradigm™? | Record of time needed to complete the assessment/ number of children who did not complete the entire paradigm | At least 80% of participants complete the entire Virtual City paradigm™ (within one hour) |
|  | **Technical smoothness** | Functioning of Virtual City ™ (sensors and software), defined as the number of technical issues and malfunctioning | Are there relevant technical issues with the paradigm (sensors, software, hardware)? | Number of participants requiring stops during the assessment due to relevant technical problems and missing data (i.e. no recording from sensors) | Less than 50% of participants |
|  | **Motivation** | Motivation and reported effort in carrying out the task | Are participants motivated to perform the task? | Number of participants that required stops due to loss of motivation and collaboration | Less than 20% of participants |
| **Feasibility of study design and procedures**  **(Virtual City ™ and neuropsychological assessments)** | **Participation willingness** | Rate of participation acceptance in the study | What is the participation rate? | Number of eligible participants that agree to join the study (both parent and child consent) | More than 80% of eligible participants |
|  | **Participation rates** | Completion of the study | Do all eligible participants, who have agreed to join the study, complete all the study procedures (Virtual City and neuropsychological)? | Number of dropouts | Less than 20% of participants who gave consensus |
|  | **Missing data** | Possibility to record all data from all outcome measures | Can all data be collected without impediments? | Total number of missing data | Less than 20% of participants with one or more missing data in one or both assessments |
|  | **Assessment time-scale** | Time required for collecting all outcome measures (within two hours and a half) | Can all data be collected within two hours and half? Either for one or two assessments | Time spent for collecting all outcome measures | More than 80% of participants complete the two assessments within a 2 and a half hour time frame |
|  | **Assessment procedure** | Failure to complete all outcome measures | Do all the participants complete the entire study? | Numbers of participants who complete both assessments | More than 80% of participants complete both assessments |
